# How elevated testosterone levels are responsible for frequent hospitalizations in female patients

**DOI:** 10.1192/j.eurpsy.2025.781

**Published:** 2025-08-26

**Authors:** S. Vuk Pisk, K. Laskarin, E. Ivezic, E. Gudelj, S. Belcic, N. Ruljancic, K. Matic, V. Grosic, I. Filipcic

**Affiliations:** 1 University Psychiatric Hospital Sveti Ivan, Zagreb; 2 Faculty of Dental Medicine and health Osijek, Osijek; 3 School of Medicine University of Zagreb, Zagreb, Croatia

## Abstract

**Introduction:**

Studies that have investigated the relationship between testosterone and psychiatric disorders in women report inconsistent results. Some studies suggesting that female depression patients have lower serum testosterone levels than healthy controls while others report higher serum testosterone levels in female depression patients. Testosterone has also been found to modulate depression and anxiety symptoms. Social research suggests high androgen levels cause aggressive behavior in men and women and as a consequence may cause depression and possible results with more often hospitalization.

**Objectives:**

The purpose of this study was to determine whether there is a link between the intensity of psychological symptoms that required hospital psychiatric treatment and the level of testosterone in the blood.

**Methods:**

The research is prospective and includes female patients with established diagnoses of depressive disorder, anxiety-depressive disorder, bipolar disorder (depressive episode) aged 18-65. The patients had their laboratory parameters determined, including sex hormones (testosteron, estrogen, progesterone, FSH, LH and prolactin), filled out a demographic questionnaire and questionnaires: The Suicide Behaviors Questionnaire-Revised (SBQ-R), Generalised Anxiety Disorder Assessment (GAD-7), Patient Health Questionnaire (PHQ-9), Beck Depression Inventory (BDI-II), Beck Anxiety Inventory (BAI), Matthey Generic Mood Question, Montgomery-Asberg Depression Rating Scale (MADRS), Hamilton Anxiety Rating Scale (HAMA) i Hamilton Depression Rating Scale (HAMD).

**Results:**

The preliminary data of the prospective study showed that there was a statistically significant proportion of patients in whom higher testosterone levels are linked with higher number of hospitalizations (rho=0.511, p=0.036).

**Conclusions:**

Preliminary results show an association between testosterone levels and frequent psychiatric hospitalizations.

**Disclosure of Interest:**

None Declared

